# Acceleration Mechanisms of Skin Wound Healing by Autologous Micrograft in Mice

**DOI:** 10.3390/ijms18081675

**Published:** 2017-08-02

**Authors:** Shiro Jimi, Masahiko Kimura, Francesco De Francesco, Michele Riccio, Shuuji Hara, Hiroyuki Ohjimi

**Affiliations:** 1 Central Laboratory for Pathology and Morphology, Faculty of Medicine, Fukuoka University, Fukuoka 8140180, Japan; 2 Department of Drug Informatics, Faculty of Pharmaceutical Sciences, Fukuoka University, Fukuoka 8140180, Japan; kim@fukuoka-u.ac.jp (M.K.); harashu@fukuoka-u.ac.jp (S.H.); 3 Department of Reconstructive Plastic Surgery-Hand Surgery, AOU “Ospedali Riuniti”, 60126 Ancona, Italy; fran.defr@libero.it (F.D.F.); michelericcio.dr@gmail.com (M.R.); 4 Departments of Plastic, Reconstructive and Aesthetic Surgery, Faculty of Medicine, Fukuoka University, Fukuoka 8140180, Japan; ohjimi@fukuoka-u.ac.jp

**Keywords:** wound healing, micrograft, granulation tissue, TGF-β1, αSMA

## Abstract

A micrograft technique, which minces tissue into micro-fragments >50 μm, has been recently developed. However, its pathophysiological mechanisms in wound healing are unclear yet. We thus performed a wound healing study using normal mice. A humanized mouse model of a skin wound with a splint was used. After total skin excision, tissue micro-fragments obtained by the Rigenera protocol were infused onto the wounds. In the cell tracing study, GFP-expressing green mice and SCID mice were used. Collagen stains including Picrosirius red (PSR) and immunohistological stains for α-smooth muscle actin (αSMA), CD31, transforming growth factor-β1 (TGF-β1) and neutrophils were evaluated for granulation tissue development. GFP-positive cells remained in granulation tissue seven days after infusion, but vanished after 13 days. Following the infusion of the tissue micrograft solution onto the wound, TGF-β1 expression was transiently upregulated in granulation tissue in the early phase. Subsequently, αSMA-expressing myofibroblasts increased in number in thickened granulation tissue with acceleration of neovascularization and collagen matrix maturation. On such granulation tissue, regenerative epithelial healing progressed, resulting in wound area reduction. Alternative alteration after the micrograft may have increased αSMA-expressing myofibroblasts in granulation tissue, which may act on collagen accumulation, neovascularization and wound contraction. All of these changes are favorable for epithelial regeneration on wound.

## 1. Introduction

Unhealed chronic wounds that develop in patients with senile decay and/or metabolic disorders including diabetes cause serious problems for not only the individual, but also society. Fast healing and successful management of the wounds become even more important today. Repair mechanisms of wound healing have been well investigated [1]; even in different organs, similar processes are involved, perhaps because of a fundamental response to injury in our bodies. Tissue response after wounding proceeds in a sequential manner [1]; after wounding by damage or tissue loss, tissue-derived factors reduce blood flow by vasodilation and initiate inflammatory cell infiltrates, which may involve tissue remodeling and a protective response against tissue infection. Granulation tissue also starts to develop as a complemental tissue at the wound site [2], in which extracellular matrix (ECM) maturation including the change in collagen type and angiogenesis/neovascularization occur. Granulation tissue is therefore important and works as a scaffold for tissue regeneration, such as epidermal regeneration in skin wounds.

After wounding, different growth factors are involved in the repair process [3], which include transforming growth factor-beta (TGF-β), platelet-derived growth factor (PDGF), connective tissue growth factor (CTGF), epidermal growth factor (EGF) and vascular endothelial growth factor (VEGF). These factors act to manifest biological roles in granulation tissue, i.e., proliferation, chemotaxis, collagen synthesis, transdifferentiating and angiogenesis/neovascularization. Granulation tissue also works for wound contraction, in which alpha-smooth muscle actin (αSMA)-expressing fibroblasts (myofibroblasts) play a central role. It is speculated that the origin of αSMA-expressing myofibroblasts is from local resident fibroblasts, bone marrow, endothelial-to-mesenchymal transition, epithelial-to-mesenchymal transition and tissue-specific mesenchymal stem cells [4,5]. Matured fat tissue also originates via dedifferentiation [6]. TGF-β1 is a portent factor for wound contraction and ECM deposition through the function of αSMA expressing myofibroblasts [5], by which ECM is remodeled in granulation tissue. At the end of wound healing, granulation tissue, including myofibroblasts, vanishes [5], and wound healing reaches its completion.

Reverdin (1869) [7] initially described the micrograft technique for wound healing, and to date, many applications with different graft sizes have been utilized [8]. Recently, an advanced and innovative micrograft technique has been established, namely the Rigenera protocol [9]. This technique embraces a unique and advantageous concept; a normal autologous tissue is minced into less than 50 μm in diameter fragments by Rigeneracons^®^. The resulting solution contains micro-tissue fragments, which also may include cellular niche and ECM in situ. Therefore, inherent cellular activity might be preserved after transplantation. Trovato et al. (2015) [9] have shown that the obtained tissue solution is rich in progenitor cells for tissue regeneration. Regarding the wound healing study, an animal study using gingival connective tissue has been recently reported [10]. Moreover, clinical trials using the technique have also been performed, such as dehiscent surgical wounds [11], complex wound [12], chronic ulcers [13] and chronic scars [14]. All of the results have demonstrated good outcomes. However, the pathophysiological mechanisms of wound healing accelerated by this technique are still unclear. Therefore, in the present study, we clarified the mechanism of action of micrograft in wound healing using our established mouse model [15].

## 2. Results

### 2.1. Minced Tissue for Micrograft

**Figure 1:**
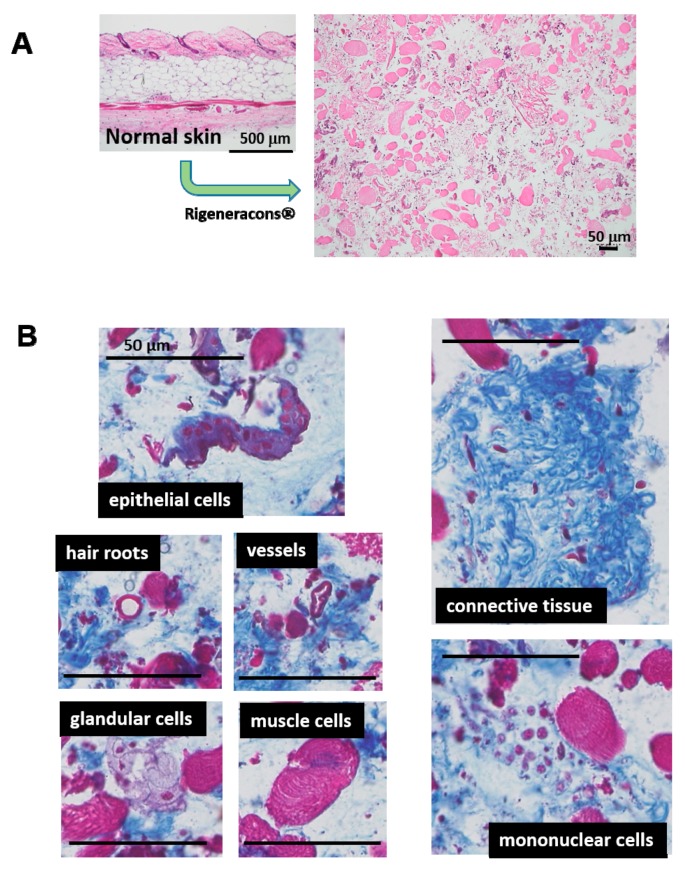
Tissue fragments in micrografted skin solution. (**A**) Normal skin tissue was minced by Rigeneracons^®^. Obtained tissues were stained with HE and observed under a microscope; (**B**) Obtained tissue was also stained with Masson’s trichrome (MT), which contained all of the skin elements, including epithelial cells, hair roots, vessels, glandular cells, muscle cells and mononuclear cells. Connective tissue is also included. Scale bars = 50 μm.

Minced skin tissue for micrograft was histologically examined. The epidermis was totally removed by scratching away with a surgical knife (Figure 1A). After mincing, tissue fragments contained all of the dermis structures, including connective tissues, muscles, fats and hair roots (Figure 1B); they were still identical to their structures. All fragments were accompanied with matrix and were about 50 μm or less in diameter.

### 2.2. Residual Micrografted Cells in Wound

**Figure 2:**
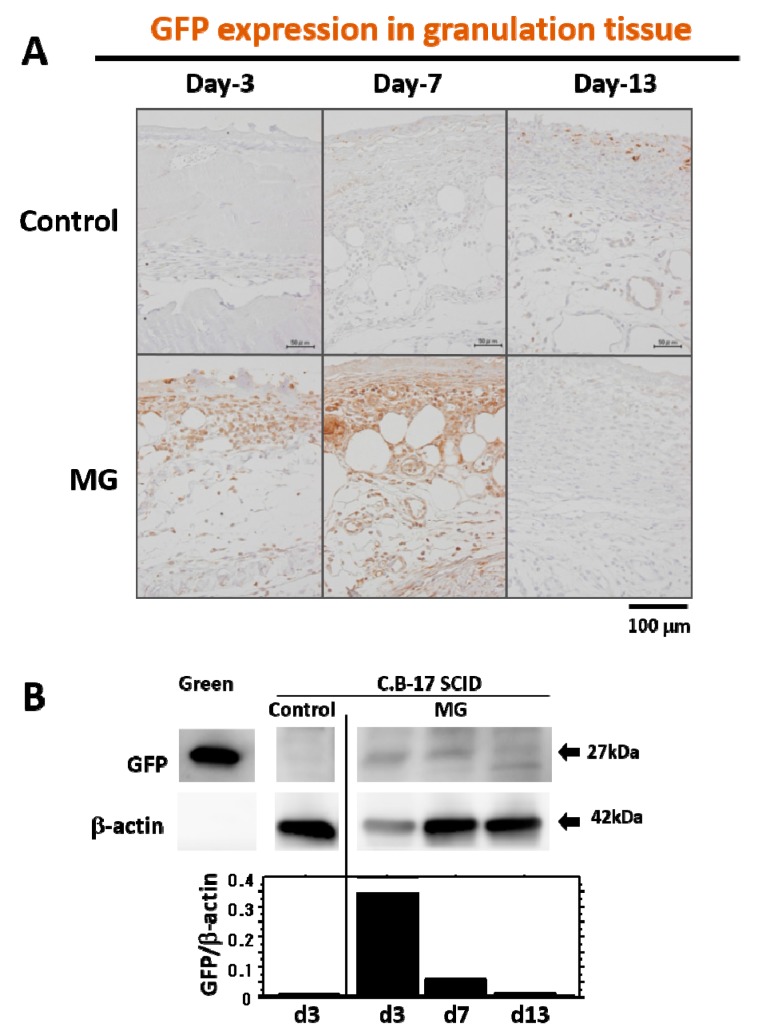
GFP expression in granulation tissue after wounding. (**A**) Presence of GFP in granulation tissue developed after wounding was immunohistochemically detected in immune-deficient SCID mice with micrograft of skin tissue solution from GFP-positive green mouse or saline alone (control) on Days 3, 7 and 13. Photographs are representative wounds in each group. Scale bars = 100 μm; (**B**) Western blot analysis for GFP in granulation tissue was performed in SCID mice with micrograft of skin tissue solution from green mouse or saline alone (control) on Days 3, 7 and 13. Green mice were used as a positive control. Densitometry analysis of GFP and β-actin was also conducted; relative expression value of GFP is shown in the bar graph.

To trace grafted cells in the wound healing process, minced skin tissue solution from green mice was inoculated on the wound surface of SCID mice just after wounding. GFP in tissue was detected using fluorescent and immunohistochemical techniques. After 3 days of inoculation, GFP cells in a round shape were found in the surficial region of immature granulation tissue (Figure 2A). On Day 7, more granulation tissue developed, and GFP-positive cells (round and spindle shape) were found to increase in matured granulation tissue (Figure 2A, Figure S1). On Day 13, in its late phase of wound healing, GFP-positive cells were scarcely found (Figure 2A). GFP protein expression was also examined with a Western blotting technique, and similar results were obtained (Figure 2B).

### 2.3. Optimal Tissue Amount for Micrograft

**Figure 3:**
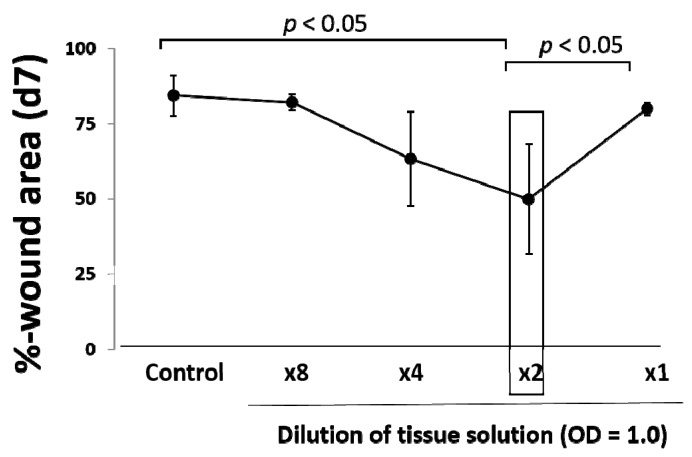
Optimal concentration of micrograft for wound healing. Optimal tissue concentration for wound healing was determined. Normal skin tissue was minced by Rigeneracons^®^, and its optical density at wavelengths of 450 nm/550 nm was adjusted to 1.0. The solution was serially diluted (2-, 4- and 8-times), and 200 μL of each solution were micrografted on the wound. Wound area was measured on seven days of the study, resulting in two-times dilution as optimal. Values: mean ± standard error (SE).

The optical density of minced skin tissue solution was measured by a microplate reader and adjusted at OD = 1.0. The optimal tissue amount to be grafted for wound healing was evaluated. After three days of wounding (mimicking clinical wounds), different amounts of tissue solution were inoculated onto the wound surface, and wound closure as %-wound area was measured on Day 7 (Figure 3). Wound area was reduced in a tissue amount-dependent manner up to the concentration of two-times dilution of OD = 1. However, no favorable effect of OD = 1 solution was found. Two-times dilution of OD = 1 (OD = 0.5) was thus used in the following studies.

### 2.4. Effects of Micrograft on Wound Healing

**Figure 4:**
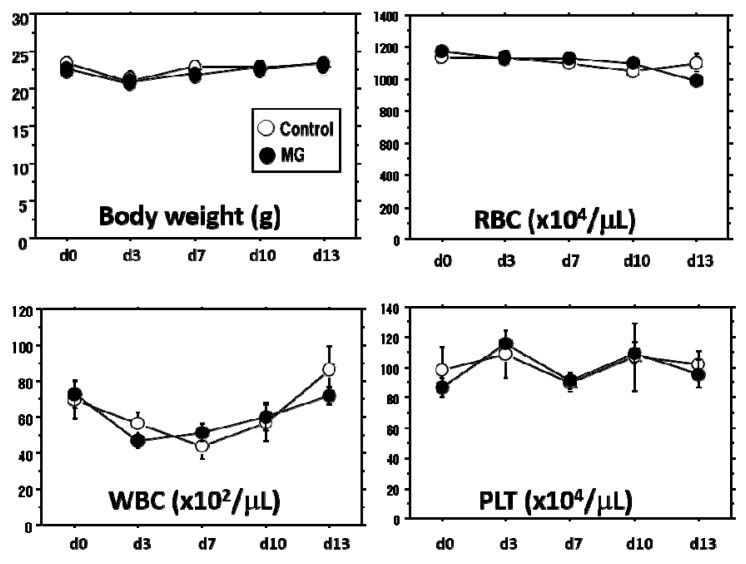
Physiological data after micrograft. The wound healing study with micrograft was performed for 13 days. Physiological monitoring (body weight) and hematological monitoring (WBC, RBC and PLT counts) were conducted. No differences were found between the MG and control groups.

**Figure 5:**
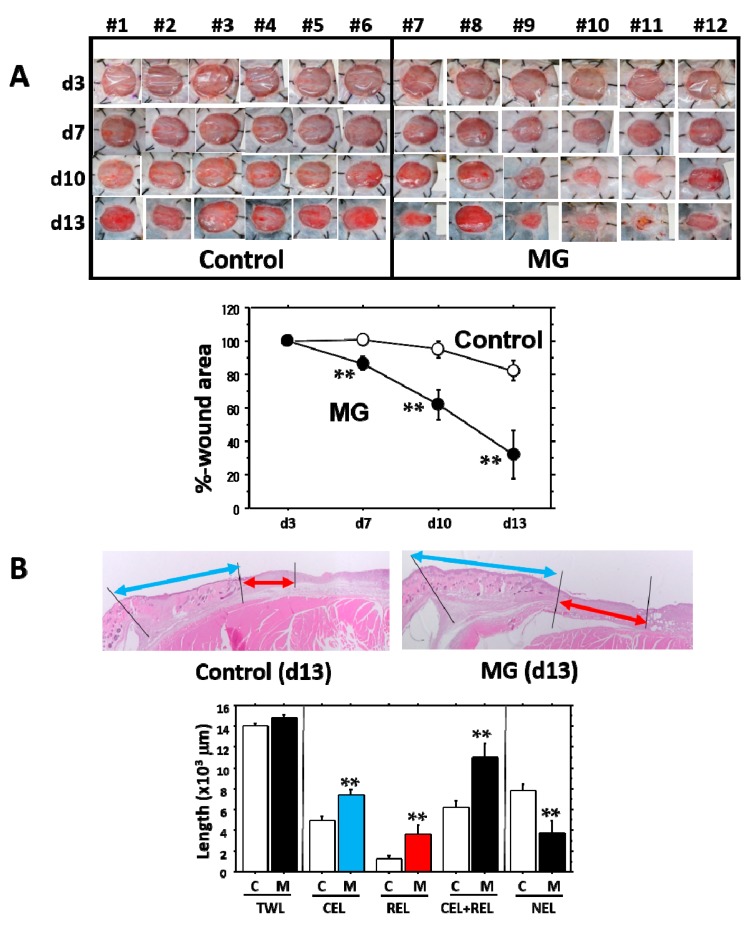
Change in wound area and histological parameters. During 13 days of the wound healing study, micrograft was performed on Day 3. (**A**) Entire wound pictures of individual mice are given. The graph shows the change of %-wound area during 13 days of the study; (**B**) The representative pictures of wound tissue 13 days after wounding in control and MG groups; epidermis shown by the blue line is in contractive healing, and epidermis shown by the red line is in regenerative healing. TWL: total wound length; CEL: contractive epithelial length; REL: regenerative epithelial length; NEL: non-epithelial length. Value: mean ± SE. **: *p* < 0.01.

In comparison with the saline control group, the effects of the micrograft were examined using C57BL/6N mice as a normal mouse. The autologous micrograft was performed on Day 3 after wounding (mimicking clinical wounds), and macroscopic wound closure and other parameters were measured up to Day 13. No difference between the groups of micrograft and saline control was found in body weight and hematological parameters including WBC, RBC and PLT during the study (Figure 4). However, healing of the macroscopic wound lesion showed a considerable difference between the groups (Figure 5A, upper panel). Wound area, recognized by its reddish color, became smaller over time in the micrograft group (MG group) accompanied with spreading of whitish tissue along the wound edge. As compared with the control, the macroscopic wound area was significantly reduced in the MG group after seven days of the study (Figure 5A, lower panel), and on Day 13, wound area in the MG group reached 30%, which was approximately three-times smaller than that of the control.

Tissue alterations especially for epithelial healing were investigated using our morphometric method previously established. Epithelialization was doubly progressed in the MG group compared with the saline control (Figure 5B); in which epidermal regenerative healing was significantly prominent in the MG group (about three-times greater than that of the control).

### 2.5. Wound Area and Granulation Tissue Thickness

**Figure 6:**
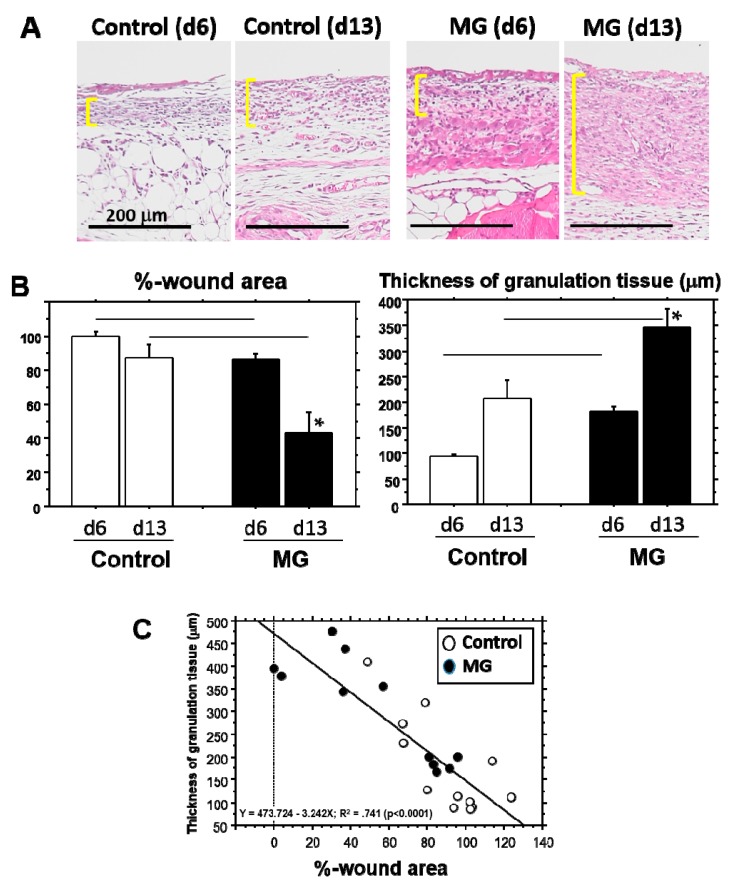
Wound area and thickness of granulation tissue. (**A**) The representative pictures of granulation tissue marked by yellow lines indicate Days 6 and 13 in control and MG groups. Scale bar = 200 μm; (**B**) %-wound area (**left panel**) and thickness of granulation tissue (**right panel**) were compared on Days 6 and 13 between control and MG groups. * *p* < 0.05 within the groups; line: *p* < 0.05 between the groups; (**C**) Regression analysis was performed between %-wound area and thickness of granulation tissue.

In a different experiment, wound areas in the MG group were again significantly smaller than that of the control group on both Day 6 and Day 13; the area in the MG group was progressively reduced during the study (Figure 6A,B). Furthermore, the granulation tissue formed beneath non-epithelialized lesion in the MG group was also significantly thicker than that of the control group on both of the days (Figure 6A,B). On Day 13, the MG group revealed doubly-thickened granulation tissue and wound area reduction as compared with the control group. Regression analysis using all of the mice showed that %-wound area and thickness of granulation tissue had a significant correlation (*p* < 0.0001) (Figure 6C).

### 2.6. Composition of Granulation Tissue

**Figure 7:**
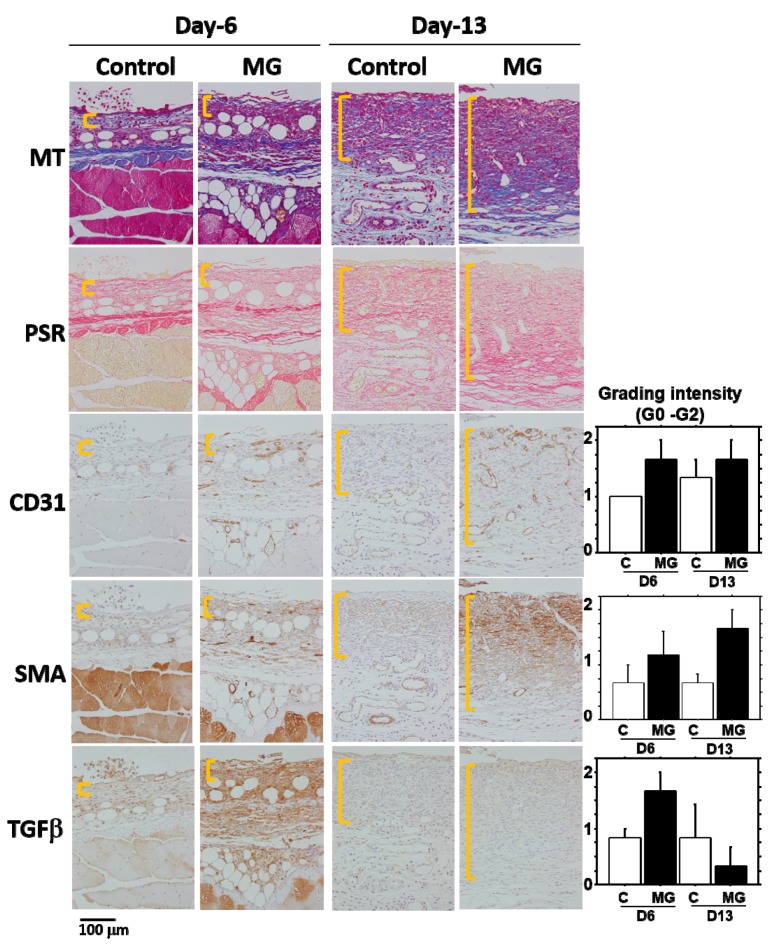
Histological alterations after micrograft. The representative pictures of granulation tissue marked by yellow lines are shown on Days 6 and 13 in the control and MG groups. Different connective tissue stains (MT and PSR) and immunohistological stains (CD31, αSMA and TGF-β1) were performed on serial sections. Scale bar = 100 μm. Inserted graphs are the semi-quantitative evaluation of CD31, αSMA and TGF-β1 in each group.

Histological evaluation was conducted on the samples used in Figure 6. The following histological results were shown as a typical result in each group at different time points. Granulation tissue, which developed beneath non-epithelialized lesion, was histologically examined. NIME-R14-positive neutrophil infiltration remained unchanged between the groups during the study (Figure S2). Collagen fibers formed in the granulation tissue stained by Masson’s trichrome (MT) and PSR (Figure 7) progressively accumulated on Day 13 and were abundant in the MG group, especially in the deeper zone. PSR stain under a polarization microscope (Figure S3) indicated that, in the MG mouse, the deeper part of the granulation tissue became red/orange in color (rich in type I collagen), while the upper part contains green color (rich in type III collagen); whereas, in the control mice, all areas of granulation tissue contain green color. Neovascularization shown by CD 31-positive endothelium took place near the wound surface on Day 6 (Figure 7). On Day 13, newly-formed capillaries proliferated in granulation tissue, which was more abundant in the MG group (Figure 7). αSMA was found in vessels, muscles and the hair outer sheath in the skin, as well as spindle cells in granulation tissue (myofibroblasts) (Figure 7). On Day 13, αSMA-positive myofibroblasts greatly accumulated in granulation tissue in the MG group (Figure 7), whereas such cells were small in number in the control group. αSMA-positive myofibroblasts were primarily distributed in the upper site of granulation tissue, where immature thin collagen fibers accumulated; alternatively, such cells were scarcely found in the deep zone with accumulation of maturated thick collagen fibers.

## 3. Discussion

An innovative micrograft technique using autologous minced tissue less than 50 μm in diameter has recently been established [9]. Many clinical trials including wound healing were already reported [11,12,13,14]; most of the results seem favorable. However, its mechanisms of action are still not clear. In the present investigation, the micrograft technique was examined using a mouse wound healing model, which we have established as a humanized wound in mice by splinting dermal tissue [15]. The method has substantial merit to estimate regenerative epithelial healing, which is important for human wound healing.

Grafted tissue was obtained from normal skin tissue, which may contain not only different cell types from different tissues (connective tissue, epidermis, hair root, vessels, glands, and so on), but also skin-originated stem cells, including epithelial stem/progenitor and mesenchymal stem cells [16]. In our tracing study of grafted tissue, the presence of GFP-expressing cells from the skin of green mice was limited in the early phase of granulation (four days after micrograft, seven days after wounding), but later on, no GFP-expressing cells were detected in matured granulation tissue. This indicates that grafted cells may be apoptosed due to completion of their initial roles for granulation tissue development, and they thus disappeared. Another possibility is that the GFP expression, which lasted for four days, may subsequently cease via alternative *GFP* gene suppression by an unknown mechanism; such cells could induce the development of granulation tissue. Molecular mechanisms of grafted cells in wound healing are still unknown. However, unlike regeneration, granulation tissue is developed as a temporary foundation tissue that supports the growth of constitutional and/or regenerative cells already present in the tissue, which subsequently vanishes after completing its roles. Therefore, this inconsecutive existence of grafted cells in granulation tissue may be reasonable in physiology.

In a previously reported study, tissue-resident progenitor cells from different human tissues were harvested by this technique, such as from the skin [13], periosteum, atrial appendage and lateral rectus muscle of the eyeball [9]. Unlike cell transplantation, normal tissue solution prepared by this technique contained not only graftable cells, but also other ungraftable cells, mechanically degenerative cells and matrix proteins. This state might mimic tissue degeneration after wounding, in which important factors for tissue repair, such as TGF-β [5], may be upregulated as seen in this study. In our results, an optimal amount of minced skin tissue for wound healing was obtained, whereas an excess amount was not favorable in outcome. This supports our hypothesis that the micrograft technique involves a mechanical tissue degenerative response, and excess degenerative manipulation thus causes imbalance in wound repair reaction, which leads to the delay of the wound healing.

It is evident that the micrograft technique accelerated skin wound healing in mice. In the study, we looked at the epithelialization responses in skin wound healing, such as CEL for contractive epithelialization from the dermal marginal zone at the cutting edge and REL for regenerative epidermis [15]. Interestingly, although CEL in the MG group showed about 1.3-times elongation versus the control group, REL elongation in the MG group greatly increased by more than three-times that of the control. This technique thereby may act primarily on regenerative healing, which is an important clinical target for human intractable ulcers. In regenerative epithelial healing, keratinocytes proliferate and migrate on granulation tissue. Both %-wound area and non-epithelialized granulation thickness were significantly greater in the MG than the control group. Remarkably, they were tightly correlated with each other. We thus focused on granulation tissue as an active supporting tissue for epidermis.

Granulation tissue is composed of infiltrated inflammatory cells, fibroblasts, newly-formed vessels and collagen matrix [1]. In our study on Day 13, collagen deposition was accelerated in the MG group, especially in the deeper zone of granulation tissue. Moreover, PSR stain showed that the deeper zone contained materials emitted that are red/orange in color (rich in type I collagen [17]), whereas the upper zone of the MG group and granulation tissue in the control group possessed green color (rich in type III collagen [17]). It has been shown that collagen type III is deposited in newly-formed/immature granulation tissue; however, normal dermal collagen primarily contains collagen type I [17]. Collagen matrix in granulation tissue could be more maturated in the MG group, which was also accompanied by an increase in neovascularization.

In the process of wound healing, wound contraction is another crucial step, in which αSMA-expressing myofibroblasts play a central role [4]. The origin of myofibroblast is thought to be derived from local resident fibroblasts through proliferation, bone marrow, endothelial-to-mesenchymal and epithelial-to-mesenchymal transition [4,5] and possibly from matured fat tissue [6]. When fibroblasts differentiate into myofibroblasts during tissue repair, αSMA is assembled in thick stress fibers [18]. Fibroblast to myofibroblast differentiation is dependent on cellular adhesion on the surrounding ECM and the presence of TGF-β1 [18,19]. Many of the myofibroblasts appeared in granulation tissue in the MG group on Day 13. Furthermore, their distribution was limited in the immature zone of granulation tissue, indicating that granulation tissue acts not only as a replenisher tissue, but also as wound contraction tissue. These are perhaps some of the reasons for the acceleration of wound healing in the MG group.

A factor responsible for the emergence of αSMA-expressing myofibroblasts is believed to be TGF-β1 in wounds [5,20]. TGF-β also stimulates collagen production [21] and neovascularization [5] and acts as an inflammatory and anti-inflammatory cytokine in different phases of wound healing [5]. In the present study, TGF-β1 was selectively accumulated in immature granulation tissue three days after the MG treatment (Day 6); and no accumulation was found on Day 13 in either of the groups. This suggests that the MG treatment may stimulate TGF-β1 production in early phase granulation, by which myofibroblast proliferation, neovascularization and collagen accumulation could be stimulated.

## 4. Materials and Methods

### 4.1. Animal Experiments

This animal study was approved by the Fukuoka University Animal Experiment Committee (No. 1507848: 3 July 2015). Study protocols were in compliance with the institution’s animal care guideline. Male C57BL/6 mice, Green mice (C57BL/6-Tg-CAG-EGFP) and CB-17SCID mice at a young age of 6–10 weeks were used in this study. All animals were purchased from Japan SLC Inc. (Shizuoka, Japan). All procedures were conducted under aseptic conditions, using autoclaves, ethylene oxide gas, 75% ethanol and povidone-iodine. Mice were anesthetized with isoflurane (Wako Pure Chemical Industries, Ltd., Osaka, Japan) or pentobarbital (Somnopentyl; KYORITSU SEIYAKU, Tokyo, Japan). Physiological checks including body weight and movement were regularly monitored. Hematological analyses, including red blood cell (RBC), white blood cell (WBC) and platelet (PLT) counts (Celltac-α, NIHON KOHDEN, Tokyo, Japan), were performed with blood collected from the orbital sinus with a heparinized 75-μL capillary (Hirschmann Laborgeräte GmbH & CO., Eberstadt, Germany). At the end of the study, the mice were sacrificed by lethal pentobarbital injection and arterial hemorrhage and wound tissue was obtained.

### 4.2. Skin Wounding

To create a humanized wound in mouse, our established splint method was used [15]. In brief, mice were anesthetized with pentobarbital, and dorsal hair was removed with a commercial depilatory. A circular tattoo (1 cm in diameter) was made at the center of the lumbar area. The part of the skin was completely excised with scissors, and a doughnut-shaped plastic splint (outer diameter: 28 mm, inner hole diameter: 18 mm) was inserted beneath the skin near the wound defect and attached to the fascia with 6-stitch ligations. The splint was then fixed to the skin with surgical silk thread (6 stitches at regular intervals). Finally, the splinted wound was covered with a polyurethane film dressing (Tegaderm, SUMITOMO 3M, Tokyo, Japan). To prevent thread removal by the mice, they were dressed and fixed with a silicon-tight vest.

### 4.3. Micrograft Using the Rigenera Protocol

Rigenera protocol established [9] was utilized. After removing the dorsal hair with a commercial depilatory, a circular tattoo (1 cm in diameter) was made on the back skin, and the marked skin was totally excised with scissors. The epidermal layer of the skin was then scraped away with a surgical knife, and the tissue was washed 2-times with saline solution. The tissue was dissected into small pieces (about 1 × 1 mm). The pieces of tissue were minced with the blade of Rigeneracons^®^ (Human Brain Wave S.r.L., Via Pinerolo, Torino, Italy). One milliliter of the tissue solution obtained through passages of the blade holes (50 μm in diameter) was used for measurement of absorbance determination (450 nm/550 nm) using a microplate reader (iMark, Bio-Rad Laboratories, Inc., Hercules, CA, USA). Tissue concentration was adjusted at OD = 1.0, and it was diluted up to 0.125-times with saline solution.

Wound surface manipulation was done as follows, unless otherwise stated. Wounds 3 days after wounding were exposed to 70% ethanol for 1 min to kill surficial cells on wounded tissue, mimicking a deterred clinical wound with necrotic tissue. After sealing the wounds with polyurethane film, 200 μL of the tissue solution using a syringe (24 G needle) were inoculated on the surface of wound by injection via peri-skin in which the tissue solution was filled in the closed space and spread over the wound surface (about 2.5 cm^2^). In the control group, saline solution alone was injected. A total of 13 days of study was performed.

### 4.4. Wound Tissue Evaluation

Macroscopic evaluation: Wound photos with scale indication were obtained from directly above with a digital camera (NEX-C3, Sony, Tokyo, Japan). The wound area was measured using a computer-assisted morphometric analyzer (VH Analyzer, VH-H1A5, KEYENCE Co., Osaka, Japan).

Microscopic evaluation: Dorsal skin tissue was dissected from the sacrificed mice. This tissue, together with the splint, was fixed in 10% buffered formaldehyde (pH 7.4) for several days. Two cross-cut tissue samples from each wound (about 5 mm thick) were excised (Sample-1 and Sample-2). Paraffin blocks were prepared by using a tissue processor (Tissue-Tec VIP Premier, SAKURA, Nagano, Japan), following which, 4-μm-thick tissue sections were cut with a microtome (RM2235, Leica Biosystems, Nußloch, Germany). For the frozen section, the Kawamoto method was used [22]. After fixing with 5% neutral formalin, tissue was embedded in super cryo-embedding medium (Leica Microsystem GmbH, Wetzlar, Germany) and then quickly frozen in liquid nitrogen. After adhesion of cryofilm (Leica Microsystem GmbH, Wetzlar, Germany) on the frozen block, a 4 µm-thick section was cut with a cryostat (CM350: Leica Microsystem GmbH, Wetzlar, Germany).

### 4.5. Histological Examination

The paraffin section was stained with hematoxylin and eosin (HE), Masson’s trichrome (MT) and Picrosirius red (PSR) [17,23]. In immunohistochemical analysis, GFP antibody (Abcam plc, Tokyo, Japan), anti-mouse αSMA antibody (Abcam plc, Tokyo, Japan), anti-mouse CD31 antibody (Dianova GmbH, Hamburg, Germany), anti-mouse neutrophil antibody (NIME-R14: Abcam plc, Tokyo, Japan) and anti-mouse TGF-β1 antibody (Abcam plc, Tokyo, Japan). An EnVision Kit (DAKO Japan Inc., Tokyo, Japan) was used for visualization. Semi-quantitative evaluation was performed: negative (Grade 0), slightly positive (Grade 0.5), positive (Grade 1) and strongly positive (Grade 2).

### 4.6. Western Blotting

Just after excision of granulation tissue in wound and sub-epidermal tissue in normal skin, tissue was sunk in RIPA lysis buffer (Merck Millipore Co., Darmstadt, Germany) containing an EDTA-free protease inhibitor cocktail (Roche Diagnostics, Indianapolis, IN, USA) and then homogenized using BioMasher (Nippi. Inc., Tokyo, Japan). Tissue lysates were then applied to a 4–15% gradient SDS-polyacrylamide gel and electrophoresed. Proteins separated in the gel were blotted onto a polyvinylidene fluoride membrane (Bio-Rad Laboratories, Hercules, CA, USA). After blocking with 5% skim milk (NACALAI TESQUE, Inc., Kyoto, Japan) in Tris buffer-saline (pH 7.6) (TBS) containing 0.1% Tween-20 (TBS-T), the membrane was reacted with primary antibodies, including anti-GFP antibody (Abcam plc, Tokyo, Japan) and anti-beta-actin antibody (Abcam plc, Tokyo, Japan). After rinsing three times with TBS-T, the membrane was incubated with a horseradish peroxidase (HRP)-conjugated secondary antibody (Cell Signaling Technology Inc., MA, USA). After washing, the membrane was incubated in ECL detection system (GE Healthcare Japan K.K., Tokyo, Japan) and luminescence was visualized by LAS-4000 mini (FUJIFILM Medical Co., Ltd., Tokyo, Japan). Densitometry was performed on targeted bands.

### 4.7. Morphometrical Analysis

Wound area percentage (WA%): Macroscopic wound evaluation was performed during the study. To obtain an accurate evaluation of wound area regression, the WA% at the time of measurement was calculated as follows: WA% = wound area at the time of measurement/wound area on Day 3 × 100.

Epithelial length from wound edge: Total epithelialization length (TEL), defined as the length of epithelial growth from the dermal cutting edge after wounding, was measured according to the previous method [15]. Two types of epithelial lengths were measured: the contracted epithelium length (CEL) and the regenerative epithelium length (REL). The TEL was the sum of the CEL + REL. The CE was characterized by normal dermal structures with hair follicles and a dermal-muscular coat, whereas the RE grown on granulation tissue did not have these characteristics.

Granulation thickness: Granulation tissue formed beneath the non-epithelialized zone was evaluated. A zonal granulation tissue (about 2000 µm in length) on the wound was selected; the length and area of the granulation tissue were morphometrically measured. The thickness of the granulation tissue was calculated by the following formula: Thickness (µm) = Area (µm^2^)/Length (µm).

### 4.8. Statistical Analysis

Values were expressed as the mean ± standard error. Linear regression analysis with least-squares estimation were done by a computer-assisted statistics program (StatView 5.0, SAS Institute Inc., Tokyo, Japan). A *p*-value of <0.05 was considered statistically significant.

## 5. Conclusions

**Figure 8:**
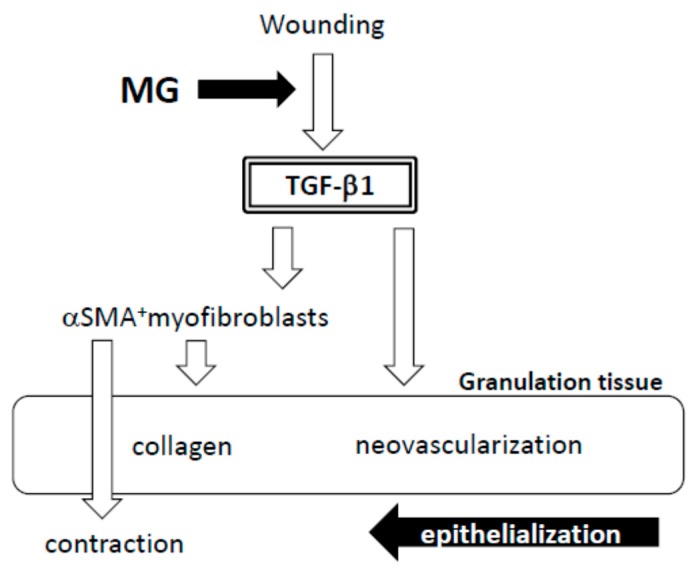
Hypothesis of wound healing by autologous micrograft.

The micrograft technique using ≤50 µm tissue fragments has been used in different clinical settings, resulting in favorable outcomes. In order to unravel its mechanism, we examined the technique to elucidate the pathophysiological mechanisms using our recently-established skin wound healing model in mouse. After micrograft of normal skin to the wound, TGF-β1 expression was selectively upregulated in granulation tissue in the early phase (Figure 8). Subsequently, αSMA-expressing myofibroblasts proliferated in thickened granulation tissue with an increase in neovascularization and collagen matrix maturation. On the basis of such granulation tissue, epithelial healing may be advanced, resulting in acceleration of the wound healing. To express cellular functions after grafting, scaffolds such as collagens may be crucially important. Thus, comparative studies with/without scaffolds should be performed in the next study. This is the first to clarify the pathophysiological mechanisms of skin wound repair response after the use of the micrograft technique. The results obtained here help to clarify the fundamental process for wound healing acceleration; as such, the effectiveness of this autologous micrograft technique may be considered for many types of intractable wounds often found in clinical practice [14].From this point of view, human wound healing treated by this technique should be investigated further. The technique is effective, easy and inexpensive, and more therapeutic applications in medical procedure and caregiving may become possible in the future.

## Supplementary Materials

Supplementary materials can be found at www.mdpi.com/1422-0067/18/8/1675/s1.

## Abbreviations

GFP	Green fluorescence protein
PSR	Picrosirius red
αSMA	Alpha-smooth muscle actin
TGF-β1	Transforming growth factor-beta1
ECM	Extracellular matrix
MG	Micrograft
%WA	%-wound area
TEL	Total epithelial length
REL	Regenerative epithelial length
CEL	Contractive epithelial length
