# Supplementary material for: Acceleration Mechanisms of Skin Wound Healing by Autologous Micrograft in Mice
Source: Int J Mol Sci. 2017 Aug 2;18(8):1675. doi: 10.3390/ijms18081675 (PMC5578065; doi:10.3390/ijms18081675)

Supplemental Figures

Supplemental Figure 1. GFP localization in granulation tissue.

Under a fluorescence microscope, GFP-positive cells in granulation tissue were found on Day 7 in the SCID mouse with the micrograft of minced skin from a Green mouse. No positive cells were found in the control mouse without micrograft.

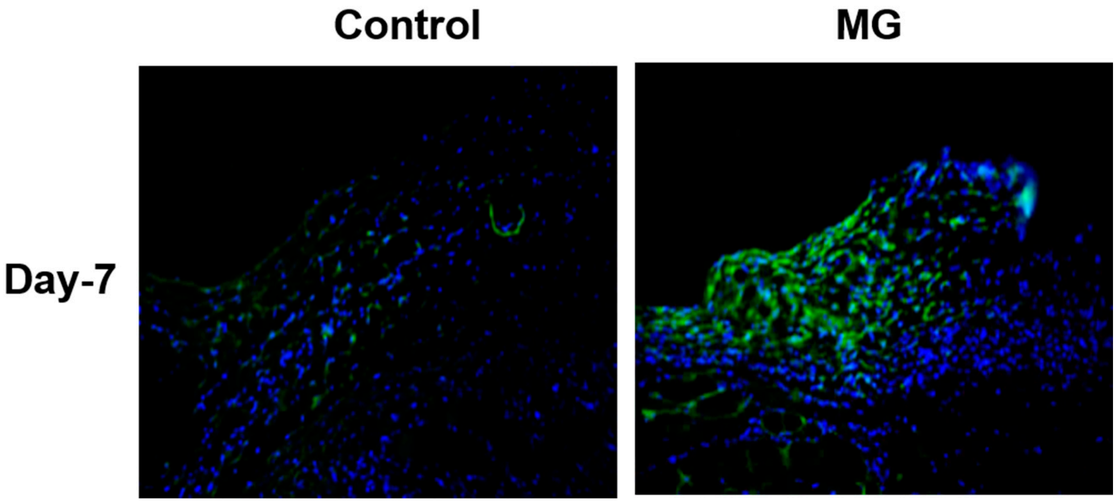

Supplemental Figure 2. Neutrophils in granulation tissue.

NIMP-R-14-positive neutrophils in granulation tissue were immunohistochemically stained on Days 6 and 13 in mice from control and MG groups. Representative pictures are shown; no difference in neutrophil density was found between the control and MG groups. However, neutrophil infiltration became more severe on Day 13 than on Day 6.

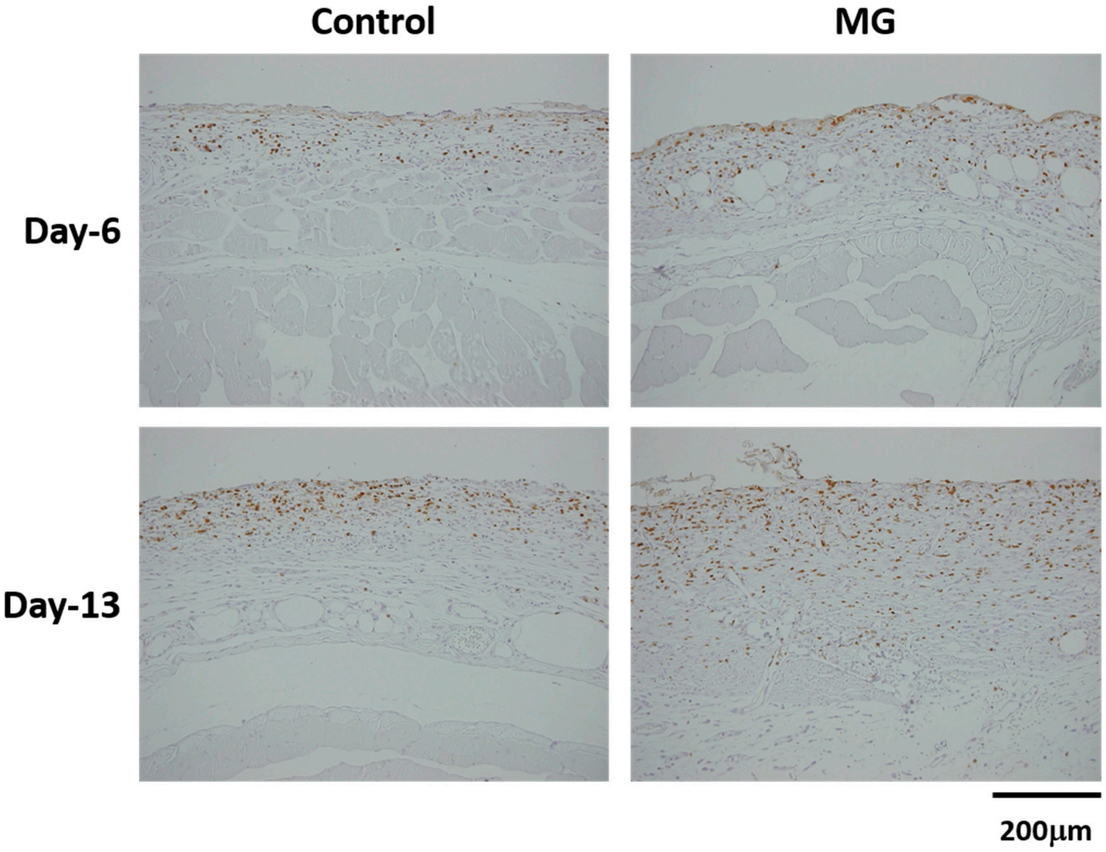

Supplemental Figure 3. Collagen deposition and αSMA-expressing myofibroblasts in granulation tissue.

Photographs are representative of granulation tissues (shown by yellow lines) in the control and MG mice on Day 13. PSR stain and its polarization view show that, in the MG mouse, the deeper part of the granulation tissue becomes red/orange in color (3: rich in type I collagen), while the upper part contains green color (2: rich in type III collagen); whereas, in the control mice, all area of granulation tissue contains green color (1). αSMA-positive myofibroblasts in granulation tissue are distributed more in the MG mouse than the control mouse. Moreover, in the MG mouse, such cells are densely accumulated in the upper part.

No. 1–3 square photos on the right: magnified sites.

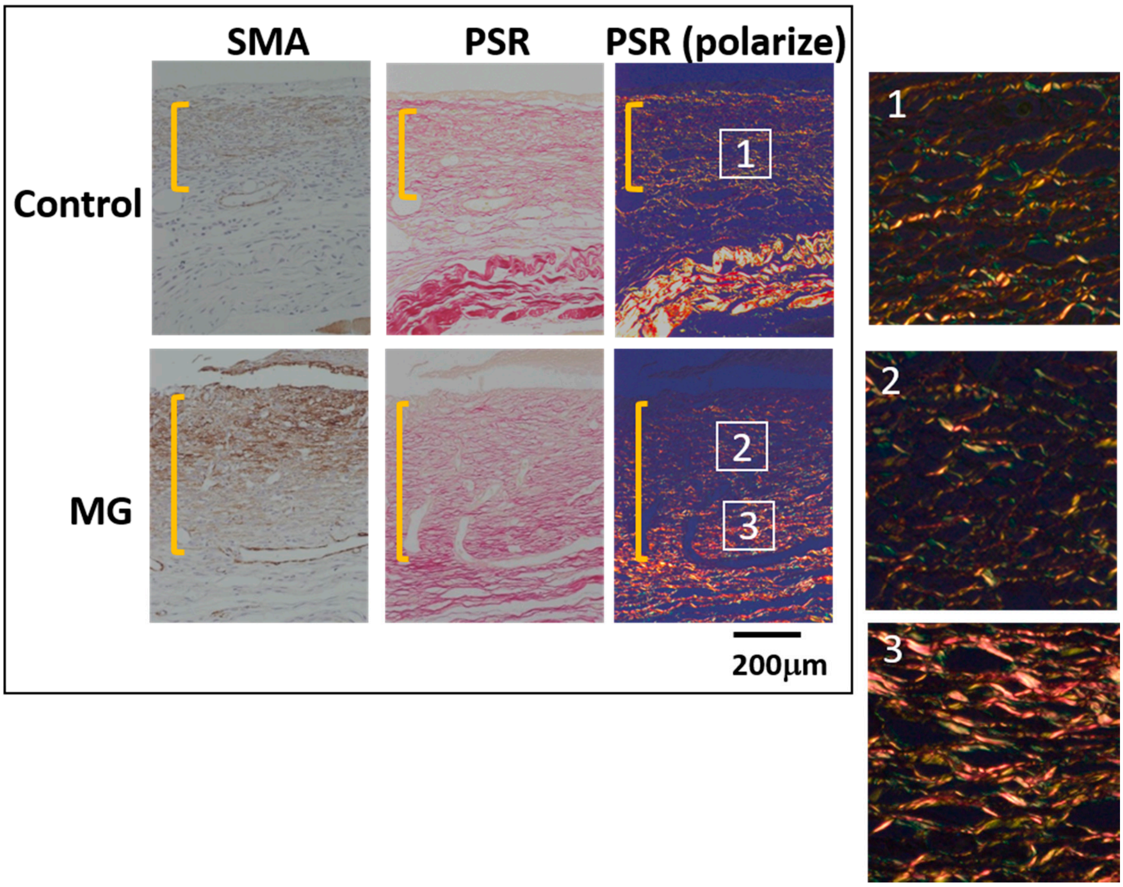

Supplement: Supplementary file 1 [file ijms-18-01675-s001.pdf]
